# Establishment of a Genetic Transformation System in Guanophilic Fungus *Amphichorda guana*

**DOI:** 10.3390/jof7020138

**Published:** 2021-02-14

**Authors:** Min Liang, Wei Li, Landa Qi, Guocan Chen, Lei Cai, Wen-Bing Yin

**Affiliations:** 1 Henan Academy of Science Institute of Biology, Zhengzhou 450008, China; liangmin202101@163.com (M.L.); 2012202024@njau.edu.cn (L.Q.); swschenggc@sina.com (G.C.); 2 State Key Laboratory of Mycology and CAS Key Laboratory of Microbial Physiological and Metabolic Engineering, Institute of Microbiology, Chinese Academy of Sciences, Beijing 100101, China; liw@im.ac.cn (W.L.); cail@im.ac.cn (L.C.); 3 College of Life Science, University of Chinese Academy of Sciences, Beijing 100049, China

**Keywords:** guanophilic fungus, genetic transformation, secondary metabolite, uridine/uracil auxotrophy, protoplast

## Abstract

Fungi from unique environments exhibit special physiological characters and plenty of bioactive natural products. However, the recalcitrant genetics or poor transformation efficiencies prevent scientists from systematically studying molecular biological mechanisms and exploiting their metabolites. In this study, we targeted a guanophilic fungus *Amphichorda guana* LC5815 and developed a genetic transformation system. We firstly established an efficient protoplast preparing method by conditional optimization of sporulation and protoplast regeneration. The regeneration rate of the protoplast is up to about 34.6% with 0.8 M sucrose as the osmotic pressure stabilizer. To develop the genetic transformation, we used the polyethylene glycol-mediated protoplast transformation, and the testing gene *AG04914* encoding a major facilitator superfamily transporter was deleted in strain LC5815, which proves the feasibility of this genetic manipulation system. Furthermore, a uridine/uracil auxotrophic strain was created by using a positive screening protocol with 5-fluoroorotic acid as a selective reagent. Finally, the genetic transformation system was successfully established in the guanophilic fungus strain LC5815, which lays the foundation for the molecular genetics research and will facilitate the exploitation of bioactive secondary metabolites in fungi.

## 1. Introduction

Fungi growing in unique environments, such as endophytic fungi, marine fungi, and coprophilous fungi, often have special physiological and metabolic characteristics [1,2,3]. Several reports have shown that endophytes are able to biosynthesize medicinally important phytochemicals and many novel bioactive natural products have also been discovered in marine fungi [4,5]. Taxol (from the endophytic fungus *Taxomyces andreanae*) and destruxins (from marine fungus *Beauveria felina*) are examples of fungi-derived compounds that are widely used in the pharmaceutical and agrochemical industries [6,7]. Coprophilous fungi are particularly rich sources of natural products given the inherent microbial competition in short-lived animal excrement [3]. They have received greater attention in recent years because of their ubiquity and ease of study [8]. A number of compounds have been isolated from them, for example, a series of new azaphilones were isolated from the goose dung-derived fungus *Coniella fragariae* [9], phytotoxic eremophilane sesquiterpenes from *Penicillium* sp. G1-a14 [10], benzophenone and fimetarone derivatives from *Delitschia confertaspora* [11], and indole alkaloids from *Aphanoascus fulvescens* [12].

Biomolecular studies are extremely important for the exploitation of these bioactive natural products. However, applications in genetic engineering are affected by the poor development of tools to be specifically employed for fungi. Although genetic transformation systems are established in some species, there is no universal transformation method for every fungal species [13,14,15]. Developing species-specific genetic manipulation methods is therefore extremely important for further molecular studies. 

*Amphichorda guana* LC5815 is a bat guano-loving fungus found in a karst cave, which is characterized by darkness, low to moderate temperatures, high humidity, and scarcity of organic matter [16]. The *Amphichorda* genus was firstly classified by Fries in 1825, and currently contains only two species, *A. felina* and *A. guana*. *A. felina* was previously classified as *B. felina* [16]. And a number of bioactive cyclodepsipeptides have been isolated from *A. felina*, including cyclosporin C and iso-isariin B [17,18], most of which have potential bioactivities including antibacterial, antifungal, insecticidal, herbicidal, and immunosuppressive properties [17,18,19]. In a preliminary study, we found that some cyclodepsipeptides (isariin A and iso-isariin B) were detected by liquid chromatography tandem mass spectrometry in strain LC5815 [17,18], however, further studies of the biosynthesis pathways in *Amphichorda* have been hindered at the genetic level, because of a lack of suitable genetic tools. In this study, we aimed to establish a genetic transformation system using the guanophilic fungus strain LC5815. We established a successful protoplast preparation method and genetic transformation system and successfully used it to delete a test gene *AG04914* (*AGMFS* (encoding a major facilitator superfamily transporter of *A. guana*)). We were also able to construct a uridine/uracil (UU) auxotrophic mutant. 

## 2. Materials and Methods

### 2.1. Strains, Materials, and Culture Conditions

Strain LC5815, strain number CGMCC 3.17908, was obtained from the China General Microbiological Culture Collection Center and used to create the genetic system in this study. Strain LC5815 and its transformants were routinely maintained on potato dextrose agar (PDA) or in potato dextrose broth (PDB) at 28 °C in the presence of appropriate antibiotics (Table 1). PDA was used as a basal medium for sporulation and protoplast regeneration. *Escherichia coli* DH5α was propagated in the Luria–Bertani (LB) medium with appropriate antibiotics for plasmid DNA isolation. The nucleotide sequence of AG04326 (AGpyrG) and AG04914 (AGMFS) are shown in the supplementary material section.

### 2.2. Maker Gene Selection and Fungal Sensitivity Test

Strain LC5815’s antibiotic sensitivity was determined in a PDA medium with varying concentrations of hygromycin B (K547, Amresco) (5, 10, 15, 20, 25, and 30 μg mL^−1^). Control plates without antibiotics were used. Sensitivity to 5-fluoroorotic acid (5-FOA) was also determined in a PDA medium with varying 5-FOA concentrations (600, 700, 800, 900, 950, and 1000 μg mL^−1^). Then, 3 × 10^5^ conidia were incubated on each plate at 28 °C for 5 days.

### 2.3. Preparation of Protoplasts

To select an efficient enzyme for protoplast preparation, we treated strain LC5815 conidia with different concentrations of Lysing enzymes (Sigma) and Yatalase (Takara) and found that the combination of 3 mg mL^−1^ of Lysing enzyme and 2 mg mL^−1^ of Yatalase was optimal for spore digestion. Strain LC5815 were grown in a PDA medium at 28 °C for 7 days, then conidia were collected with 0.1% (*w/v*) Tween-80 water and sterile cotton filters. Conidia suspensions were centrifuged at 5000 rpm for 10 minutes (min), the supernatants aspirated, and the conidia were resuspended in a PDB medium for spore germination. Conidia were incubated in 30 mL PDB medium in a 100 mL Erlenmeyer flask at 28 °C with shaking at 200 rpm for about 28 h. Germinated conidia pellets were collected in 50 mL centrifuge tubes in a bench-top centrifuge, at 5000 rpm for 5 min at room temperature. Germinated conidia were washed twice with sterile H_2_O to remove medium residue then gently resuspended in 10 mL osmotic medium [21] with 30 mg Lysing enzymes and 20 mg Yatalase. The conidia were incubated at 28 °C for about 4 h with shaking at 150 rpm. The first microscopic observation was at 3 hours, then at 15 min intervals until homogeneous protoplasts, about twice the size of spores, were formed. Protoplasts suspension were transferred into a Falcon tube and overlaid with 10 mL trapping buffer (0.6 M sorbitol, 100 mM Tris/HCl, pH = 7.0) extremely carefully. Protoplasts were taken from the interface with a bent glass pipette and then added STC (0.01 M Tris/HCl pH 7.5, 0.01 M CaCl_2_, and 1.2 M sorbitol) buffer (2-2.5 times of protoplast volume) into 15 mL Falcon tubes. Finally, protoplasts were collected by centrifuge at 5000 rpm and 4 °C for 15 min.

### 2.4. Regeneration of Protoplasts

Protoplast pellets were resuspended in 1 mL STC buffer and centrifuged at 13,000 rpm and 4 °C for 20 s, then put into 1.5 mL tubes; the concentration of protoplasts was adjusted to 10^8^ mL^−1^ through a hemocytometer. PDA with different osmotic stabilizers including KCl (0.8 M), NaCl (0.8 M), sucrose (0.8 M), and sorbitol (1.2 M) was used as a regeneration medium. The 10^8^ mL^−1^ protoplast solution containing osmotic pressure stabilizers was diluted to different concentrations (10^5^ mL^−1^, 10^4^ mL^−1^, 10^3^ mL^−1^) using STC buffer, then 500 μL was inoculated onto the surface of the regeneration medium, allowing us to study the effects of osmotic stabilizers on the regeneration frequency of strain LC5815 protoplasts. PDA medium without osmotic pressure stabilizers was used as control, and a protoplast solution with water was used as a negative control. Protoplasts were cultivated at 28 °C for about 3 days, and single colonies of regenerated protoplasts were counted on the Petri dishes using microscopy.

### 2.5. Construction of Deletion Cassettes

The plasmid pAG1-H3 containing the hygromycin phosphotransferase gene (*hph*) was used in this study (Table 1). The oligonucleotide sequences of PCR amplification primers used in this study are given in Table 2. To create deletion strains of AG04914 (AGMFS) and AG04326 (AGpyrG), we applied the double-joint and single-joint PCR method to construct the knockout cassette [22]. Approximately 1.4 kb fragments upstream and downstream of the target gene *AG04914* were amplified from genomic DNA of strain LC5815 using designated primers (Figure S1 and Table 2). The *hph* marker fragment was amplified from pAG1-H3 using the primers shown in Table 2. The three *AG04914* fragments and the *hph* marker fragment were purified with an EasyPure Quick Gel Extraction Kit (Transgene Biotech). The purified fragments were quantified and assembled using double-joint PCR to create the *AG04914* deletion cassette. The two *AG04326* fragments were assembled by single-joint PCR to create the *AG04326* deletion cassette (Figure S3).

### 2.6. PEG-Mediated Protoplast Transformation

Ten micrograms of deletion cassettes were gently mixed with 10 μL of STC buffer then added to 100 μL of protoplasts containing at least 10^8^ protoplasts per mL and incubated on ice for 50 min. Then, 1.25 mL of 60% polyethylene glycol (PEG) (PEG 6000 0.6 g mL^−1^, CaCl_2_·2H_2_O 7.35 mg mL^−1^, 10 mM Tris-HCl, pH = 7.5) was added to the mixture and gently mixed then incubated at room temperature for 30 min. Five milliliters of STC was added and gently mixed, after which the mixture was divided into five equal aliquots to uniformly distribute on the selection medium plates (SPDA (PDA supported with 0.8 M sucrose) with the selection marker). Five milliliters of warm top regeneration medium (containing a selection marker) was immediately added to the plates to osmotically embed the protoplasts [21]. Untransformed protoplasts were added to control plates with and without antibiotic. All plates were incubated at 28 °C for 5–7 days. 

### 2.7. Verification of Transformants

Transformants were streaked out twice to obtain single colonies in a PDA medium with antibiotics to ensure their mitotic stability. Selected transformants were grown in a liquid PDB medium for genomic DNA extraction was carried out according to a previously described protocol [23]. The selected mutants were tested by diagnostic PCR using primers inside and outside of the corresponding gene, as listed in Table 2.

## 3. Results

### 3.1. Maker Gene Selection and Fungal Sensitivity

**Figure 1:**
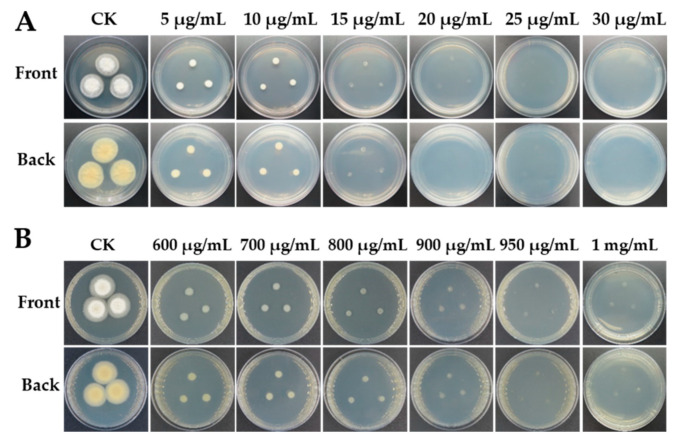
Sensitivity of wild type strain LC5815 in the PDA medium of hygromycin B (**A**) and 5-FOA (**B**) at 28 °C for 5 days, CK: culture without additions of chemicals.

A transformation system requires a selective agent that can be used to differentiate transformed isolates from untransformed ones. To identify useful agents for the selection of strain LC5815 transformants, strain LC5815 sensitivity to hygromycin B was used as a selectable marker. Strain LC5815 growth was completely inhibited at 20 μg mL^−1^ hygromycin B (Figure 1A). To construct the UU auxotroph strains, strain LC5815 sensitivity to 5-FOA was tested, and it was found that the strain was completely inhibited at a concentration of 1 mg mL^−1^ (Figure 1B). Based on these sensitivities, the *hph* gene was chosen as the marker gene, and 1 mg mL^−1^ 5-FOA was used to construct the UU auxotroph.

### 3.2. Protoplast Preparation and Regeneration of Strain LC5815

The fungal cell wall with its complex composition is the most important problem to be considered when preparing protoplasts. In this study, two cell wall digesting enzymes, Lysing enzyme and Yatalase, were combined to test protoplast release from strain LC5815 conidia. In terms of economy, time, and efficiency, results showed that 3 mg mL^−1^ Lysing enzyme and 2 mg mL^−1^ Yatalase were the optimal concentrations to use for protoplast release from strain LC5815. Protoplast quality is influenced by multiple factors including enzymolysis temperature and duration [24,25]. No obvious differences were seen in the number of released fungal protoplasts when the digestion reactions were incubated at temperature 25 °C or 28 °C. We observed that incubating conidia with Lysing enzymes and Yatalyse could yield about 3.0 × 10^8^ protoplasts after incubation at 150 rpm for about four hours. This yield is enough to perform 20 transformation experiments.

**Figure 2:**
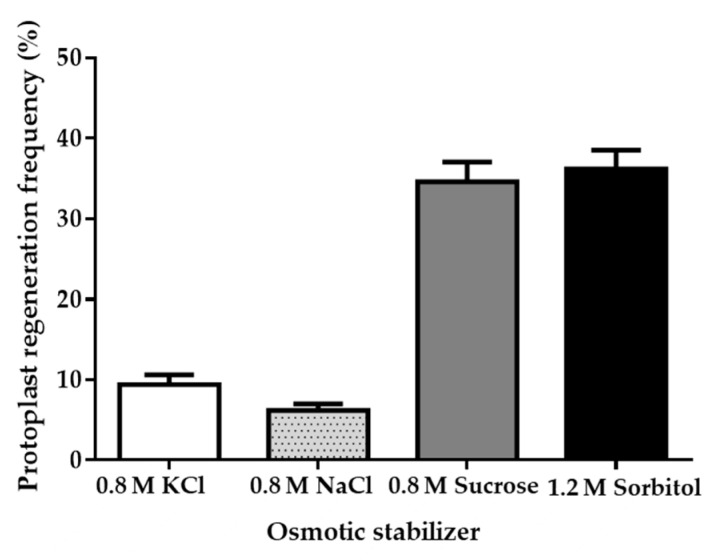
Effects of osmotic pressure stabilizer on strain LC5815 protoplast regeneration rate.

The incubation environment influences the isolation and regeneration of fungal protoplasts, and osmotic pressure stabilizers play an important role here [26]. Four different osmotic pressure stabilizers (0.8 M NaCl, 0.8 M KCl, 0.8 M sucrose, 1.2 M sorbitol) were tested for protoplast regeneration in a PDA regeneration medium [27]. We chose a PDA medium containing various osmotic pressure stabilizers to analyze the regeneration rate, with the optimized protoplast formation system. The regeneration number of diluted protoplasts (0.5 × 10^3^) can be calculated using microscopy. Results indicate that 0.8 M sucrose and 1.2 M sorbitol had the better effect and led to a protoplast regeneration rate of nearly 40% (Figure 2). Taking economy and efficiency into consideration, we found that 0.8 M sucrose is more suitable as the osmotic pressure stabilizer for strain LC5815 protoplast regeneration.

### 3.3. PEG-Mediated Protoplast Transformation and Target Gene Deletion

**Figure 3:**
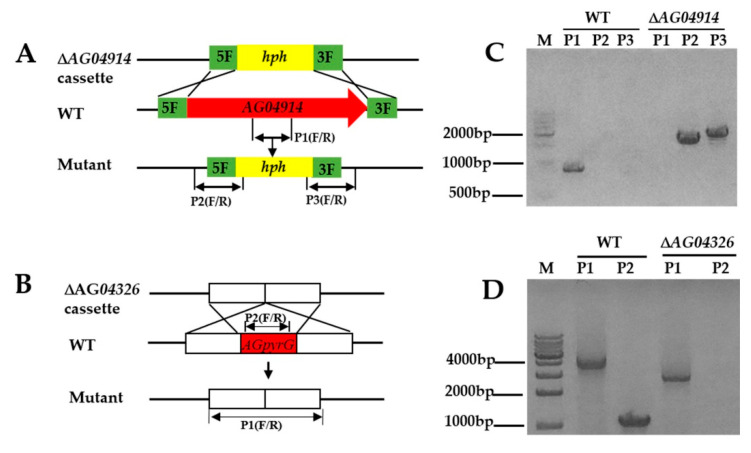
Schematic illustration of deletion and confirmation of strain LC5815 mutants. (**A**) Strategy for homologous recombination of strain LC5815 for *AG04914* gene disruption using a *hph* gene as a selectable marker. (**B**) Scheme of the destruction of the *AGpyrG* locus in the parental strain LC5815 by homologous recombination yielding a *AGpyrG* deletion strain. (**C**) Diagnostic PCR to identify the Δ*AGpyrG* mutant with three primer pairs (P1, P2, and P3). (**D**) Diagnostic PCR to identify the Δ*AGpyrG* mutant with two primer pairs (P1 and P2).

**Figure 4:**
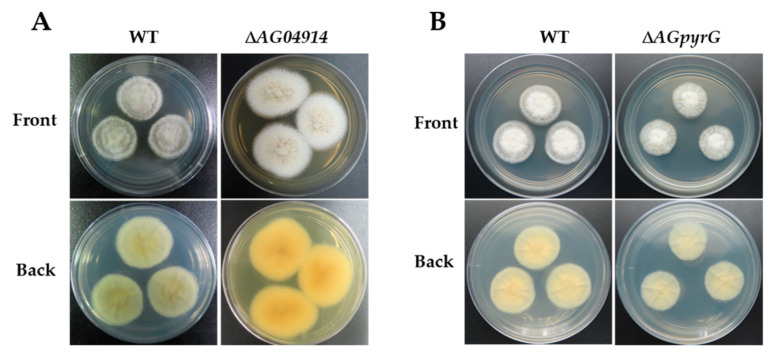
Morphological comparison between the strain LC5815 wild type and mutants. (**A**) The phenotypes of the strain LC5815 wild type and Δ*AG04914* mutant on PDA plate at 28 °C for 7 days. (**B**) The phenotypes of the strain LC5815 wild type and Δ*AG04326* mutant on PDA plate without or with UU at 28 °C for 5 days.

The protoplast transformation efficiency was tested using the deletion of *AG04914*, a gene that encodes one protein belonging to a major facilitator superfamily (MFS) transporter and consists of 427 amino acids. AG04914 has a high sequence identity in other Ascomycetes (66% in *Coniochaeta ligniaria* NRRL 30616 (OIW33124.1); 68% in *Lophiotrema nucula* (KAF2109962.1)). Protoplasts without a knockout cassette (Δ*AG04914*) were inoculated in bottom medium with hygromycin B (20 μg mL^−1^) as control. Fifteen single colonies grew in the selective plate while no colonies were seen in the control plate. The colonies were then grown in a resistant medium, and nine single colonies grew in the hygromycin B plate. Further identification of the resistant colony was obtained by diagnostic PCR (Figure S2). Finally, one correct mutant was obtained (Figure 3C and Figure 4A). The data here showed that non-homologous end joining may block the integration rate at the homologous site [28].

Meanwhile, to enable us to use strain LC5815 to perform extensive genetic manipulation, we constructed a UU auxotroph mutant. Firstly, the homologous genes of *pyrG* from the strain LC5815 genome were obtained by homologous Basic Local Alignment Search Tool (BLAST). The protein sequences of pyrG from model fungi including *Aspergillus nidulans* and *A. fumigatus* were used as queries for BLAST searches. The predicted strain LC5815 AGpyrG protein consists of 369 amino acids and shares high sequence similarity with pyrG from other Ascomycetes (50% in *A. nidulans* (AN6157.2); 48% in *A. fumigatus* (CAA72161.1)). To generate UU auxotrophic mutants, the *AG04326* deletion cassette was transformed into the strain LC5815 protoplasts using the homologous recombination strategy. As expected, the WT strain was sensitive to 5-FOA, whereas the knockout *AG04326* strain was resistant to 5-FOA in the presence of UU. The transformants were cultivated in a selective PDA medium supplemented with 0.5% uridine, 0.5% uracil, and 1 mg mL^−1^ of 5-FOA. To verify these mutants, genomic DNA was extracted, and diagnostic PCR was performed using the designated primers. In the correct mutants, a band of 2.7 kb was seen, while in the WT strain, a 3.9 kb band was seen, showing that it contained a 1.2 kb fragment of the *AGpyrG* gene. The *AG04326* gene fragment could not be amplified in the mutants (Figure 3D and Figure S4). The results show that the UU auxotrophic mutant was successfully created and cannot grow in a normal PDA medium. However, the UU auxotrophic mutant grew at a significantly slower rate in a PDA medium with UU compared to the WT strain in a PDA medium without UU when they grew for five days at 28 °C (Figure 4B).

## 4. Discussion

Genetic manipulation has played a major role in the mining and development of natural products. Transformation technology is the basis of genome modification in filamentous fungi. *Agrobacterium tumefaciens*-mediated transformation and PEG-mediated protoplast transformation are the two commonly used systems in filamentous fungi [15,29]. *A. tumefaciens*-mediated transformation is affected by many factors, such as types of *Agrobacterium* strains, higher efficiency vector, and co-culture conditions. As it may also interrupt the original genes of the host or cause chromosome rearrangement, we used PEG-mediated genetic transformation to establish a genetic transformation system in guanophilic fungus strain LC5815 [30,31,32].

Most of the current gene manipulation techniques depend upon efficient protoplast isolation and regeneration [33]. In this process, osmotic pressure stabilization is important for maintaining and controlling protoplast numbers. When internal and external pressures are the same, the mycelium is in a stable physiological state and protoplasts are released smoothly and completely. Different osmotic pressure stabilizers have a considerable influence on the protoplast yield. If the osmotic pressure stabilizer concentration is too high, or too low, protoplast preparation and regeneration can be severely affected. The osmotic pressure stabilizer also affects enzyme activity, which indirectly affects protoplast yield [24,34]. In this study, we optimized the spore germination medium and time for strain LC5815. We also determined the appropriate lyase and incubation time to prepare the protoplasts and the optimal osmotic pressure stabilizer (0.8 M sucrose) for genetic transformation.

Marker genes play an important role in fungal genetic transformation, and increasing the number available will greatly facilitate the study of secondary metabolite biosynthesis. Hygromycin B phosphotransferase gene (*hph*) and neomycin phosphotransferase gene (*neo*) (G418 resistance) are two commonly used antibiotic transformation markers. Construction of a UU auxotroph has also been widely shown to be efficient in the genetic transformation of Ascomycetes [35,36,37]. *PyrG* in *Aspergillus* (*pyr4* in *Trichoderma*, *ura3* in *Mortierella*) encodes an orotidine 5’-monophosphate (OMP) decarboxylase, which can convert exogenous 5-FOA into a toxic intermediate. Therefore, by knocking out *pyrG* the mutant can survive on 5-FOA-containing medium supplemented with exogenous UU, and *pyrG* can also be used as a marker gene for complementation. *Amphichorda* spp. represent a rich production source for bioactive cyclodepsipeptides which are widely used in the pharmaceutical and agrochemical industry [7,17,18,38]. The UU auxotroph created in this study will allow us to target and modify genes efficiently in this rare genus.

Currently, there are some genetic transformation methods for filamentous fungi, and many factors affect their efficiency [39]. Here, we optimized some key factors for genetic transformation and reported basic protocols to prepare and regenerate protoplasts of a special *Amphichorda* strain. Because different fungi generally have unique and complex cell wall structures, it is not clear whether our method can be applied to other fungal species. Other genetic transformation methods will also need to be studied and compared to see whether they are more effective in this rare genus.

## 5. Conclusions

In conclusion, we developed an improved PEG-based method for the genetic transformation of strain LC5815 of the rare guanophilic species *A. guana*. We also demonstrated that the *hph* marker gene can be efficiently used for strain LC5815. The feasibility of this transformation system was proved by the deletion of an MFS transporter and the creation of a UU auxotrophic strain. Our study will provide access to study the relevant molecular biological mechanism and exploit novel secondary metabolites in *Amphichorda*.

## Figures and Tables

**Table 1:** Strains and plasmids used in this study.

Strain/Plasmid	Description	Reference
*A. guana* LC5815	Wild type (WT, CGMCC 3.17908)	[16]
pAG1-H3	Hygromycin resistance vector	[20]
TYML6	Δ*AG04914*::*hph* in WT	This study
TYML7	Δ*AG04326*::*hph* in WT	This study

**Table 2:** PCR primer sets used in this study.

Primers	Oligonucleotide Sequence (5′-3′)	Uses
HYG_F	atcgatgatcaggcctcgac	Maker hygromycin amplification
HYG_R	gtgcattctgggtaaacgactc	Maker hygromycin amplification
HYG_RT_R	ccgagagctgcatcaggtc	Δ*AG04914* transformant 5F screening
HYG_RT_F	gcgaagcagaagaatagcttagc	Δ*AG04914* transformant 3F screening
KO04326-5F-F	gtgcatcgcagcgattgatg	Up flanks’ amplification for *AG04326* deletion
KO04326-5F-nest	cgccaggctaatgcactatg	Diagnostic PCR for *AG04326* deletion
KO04326-5F-R	cactcatcacagcttgtcgatcccgatgctgaatcggtag	Up flanks’ amplification for *AG04326* deletion
KO04326-3F-F	cgattcagcaatcgggatcgacaagctgtgatgagtgcag	Down flanks’ amplification for *AG04326* deletion
AGpyrG-3F-R-nest	ctggtagttgaaggtggtcagg	Diagnostic PCR for *AG04326* deletion
AGpyrG-3F-R	gtacttgagcgcctcgaactc	Down flanks’ amplification for *AG04326* deletion
C35-04914-5F-F	ccgaattgaccaggtgcttc	Δ*AG04914* transformant 5F screening
C35-04914-5F-nest	ggccagtcgtacttgtagacg	knockout *AG04914* cassette
C35-04914-5F-R	gtcgtttacccagaatgcacggatcacaccgagaagagcag	knockout *AG04914* cassette
C35-04914-3F-F	gtcgaggcctgatcatcgatcggatctcctcagtacatggc	knockout *AG04914* cassette
C35-04914-3F-nest	gcctctgttgttgccattgtc	knockout *AG04914* cassette
C35-04914-3F-R	gatctctccgctgaggaacag	Δ*AG04914* transformant 3F screening

## Data Availability

The data presented in this study are available in this manuscript, and constructs can be requested from the corresponding author.

## Supplementary Materials

The following are available online at https://www.mdpi.com/2309-608X/7/2/138/s1.
