# Supplementary material for: Establishment of a Genetic Transformation System in Guanophilic Fungus Amphichorda guana
Source: J Fungi (Basel). 2021 Feb 14;7(2):138. doi: 10.3390/jof7020138 (PMC7918455; doi:10.3390/jof7020138)
Supplement: Supplementary file 1 [file jof-07-00138-s001.pdf]

Original Images for Gels

Establishment of a genetic transformation system in guanophilic fungus *Amphichorda guana*

Min Liang [1,2,3†], Wei Li [2,†], Landa Qi [1], Guocan Chen [1], Lei Cai [2] and Wen-Bing Yin [2,3*]

[1] Henan Academy of Science Institute of Biology, Zhengzhou 450008, China; liangmin202101@163.com (M.L.); 740279714@qq.com (L.Q.); swschenggc@sina.com (G.C.)

[2] State Key Laboratory of Mycology and CAS Key Laboratory of Microbial Physiological and Metabolic Engineering, Institute of Microbiology, Chinese Academy of Sciences, Beijing 100101, P.R. China; liangmin202101@163.com (M.L.); liw@im.ac.cn (W.L.); cail@im.ac.cn (L.C.)

[3] University of Chinese Academy of Sciences, Beijing, 100049, China; liangmin202101@163.com (M.L.)

* Correspondence: Wen-Bing Yin, E-mail: yinwb@im.ac.cn. Tel: 86-10-64806170.

† These authors contributed equally to this work.

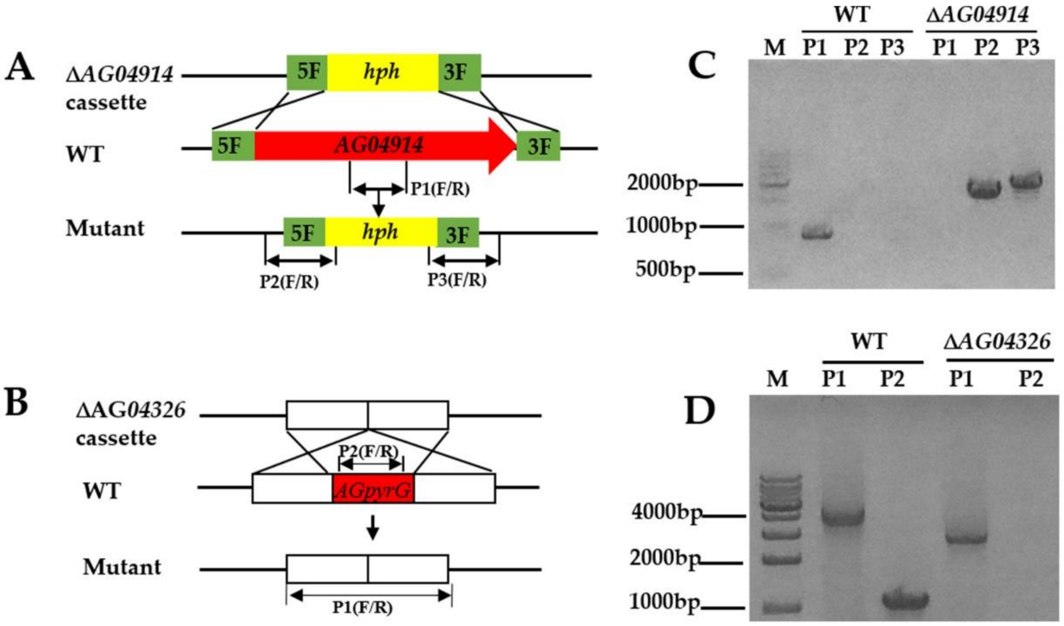

Figure 3. Schematic illustration of deletion and confirmation of *A. guana* mutants. (**A**) Strategy for homologous recombination of *A. guana* for *AG04914* (*AGMFS*) gene disruption using a *hph* gene as selectable marker. (**B**) Scheme of the destruction of the *AG04326* (*AGpyrG*) locus in the parental strain *A. guana* by homologous recombination yielding a *AG04326* (*AGpyrG*) deletion strain. (**C**) Diagnostic PCR to identify the Δ*AG04914* (*AGMFS*) mutant with three primer pairs. (**D**) Diagnostic PCR to identify the Δ*AGpyrG* mutant with two primer pairs (P1 and P2).

Original Images of Figure 3A and 3C for Gels

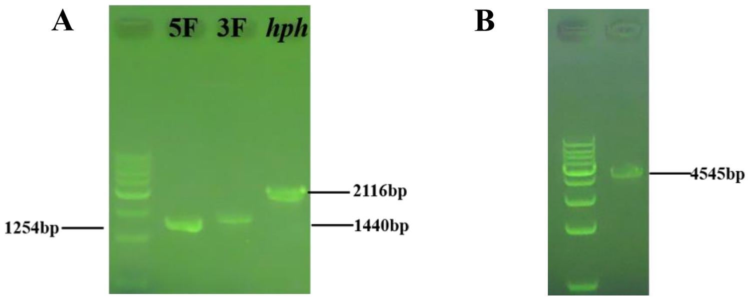

Figure S1. Construction of deletion cassette of *AG04914* (*AGMFS*) by Double-joint PCR. (**A**) Upstream and downstream of the target genes *AG04914 (AGMFS)* were amplified from genomic DNA of *A. guana* LC5815 using designated primers, respectively (Table 2). The *hph* marker fragment were amplified from pAG1-H3 using appropriate primers in Table 2. (**B**) These three purified PCR fragments of *AG04914* (*AGMFS*) and *hph* marker fragment were purified were assembled to yield the *AG04914* (*AGMFS*) deletion cassette.

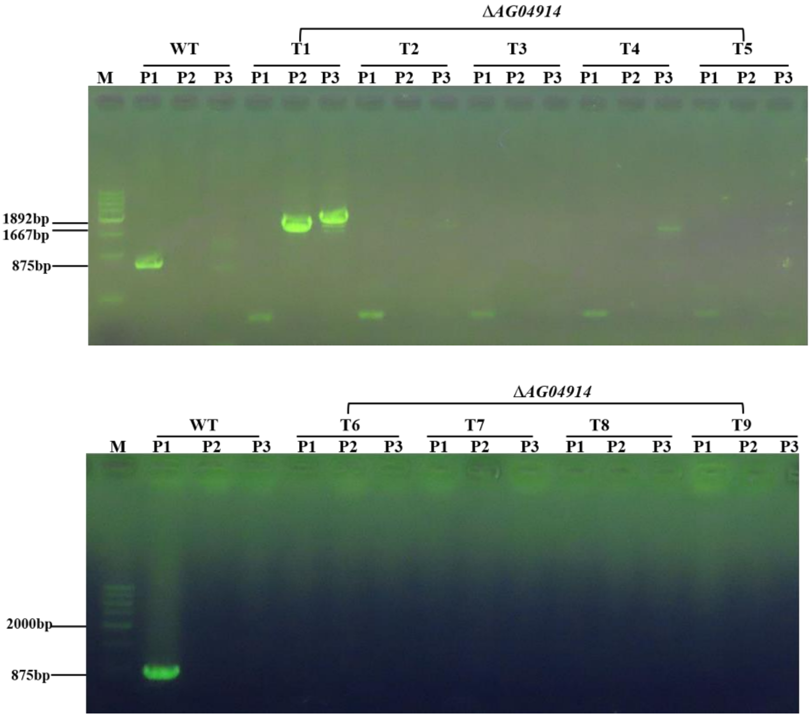

Figure S2. Diagnostic PCR to identify the Δ*AG04914* (*AGMFS*) mutant with three primer pairs.

Original Images of Figure 3B and 3D for Gels

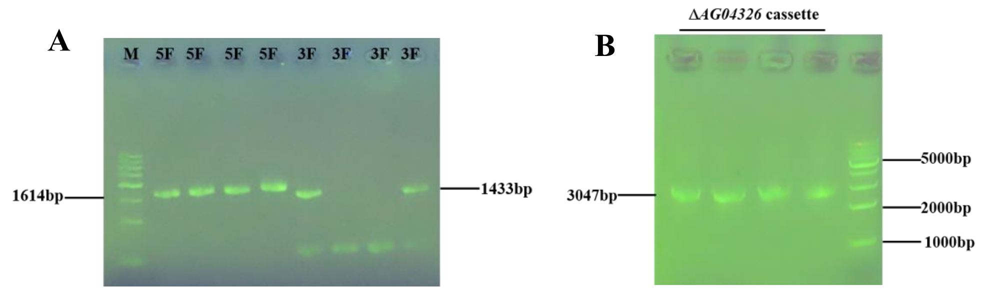

Figure S3. Construction of deletion cassette of *AG04326* (*AGpyrG*) by Single-joint PCR. (**A**) Upstream and downstream of the target genes *AG04914 (AGMFS)* were amplified from genomic DNA of *A. guana* LC5815 using designated primers, respectively (Table 2). (**B**) Two purified PCR fragments of *AG04326* (*AGpyrG*) were purified and assembled to yield the *AG04326* (*AGpyrG*) deletion cassette.

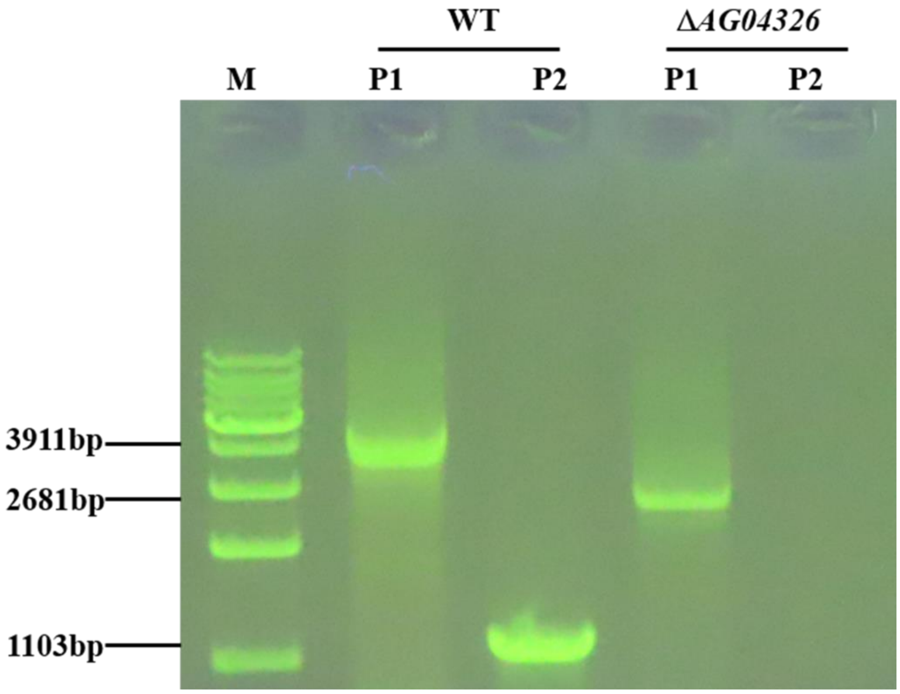

Figure S4. Diagnostic PCR to identify the *AG04326* (*AGpyrG*) mutant with two primer pairs.
